# Taming the chaos ?! – part 2: limitations of using eXplainable Artificial Intelligence (XAI) to tackle the complexity in psychotherapy of children and adolescents

**DOI:** 10.1007/s00787-024-02487-7

**Published:** 2024-06-07

**Authors:** Veit Roessner, Anne Uhlmann, Stefan Ehrlich, Robert Waltereit

**Affiliations:** 1 Department of Child and Adolescent Psychiatry, Technische Universität Dresden, Fetscherstrasse 74, 01307 Dresden, Germany; 2 Division of Psychological and Social Medicine and Developmental Neurosciences, Translational Developmental Neuroscience Section, Faculty of Medicine, Technische Universität Dresden, Fetscherstrasse 74, 01307 Dresden, Germany; 3 Department of Child and Adolescent Psychiatry, LWL-Klinikum Marsberg, Bredelarer Strasse 33, 34431 Marsberg, Germany

Working in the field of child and adolescent psychiatry, also referred to as child and adolescent psychiatry and psychotherapy in some countries, necessitates consideration of a multitude of variables. These variables depend on a vast range of factors (e.g. age, gender, cognitive abilities, family situation, culture) in each individual case and are intricately linked, creating a landscape of immense complexity. This complexity further arises from factors such as the individual impact of developmental stages, cause-and-effect relationships, and mediating and moderating influences. Additional complications lie in the subjective nature of many variables [note: in child and adolescent psychiatry, there are only two (ICD-10) or one (ICD-11) objective, reliable and verifiable criteria: for the diagnosis of anorexia amenorrhea (yes/no) and body weight (in kg)]. Discrepancies can arise between a child’s self-perception and their parents’ perspective, or even between the judgments of each parent in high-conflict situations. Despite these challenges, significant strides have been made in understanding the classification, diagnosis, etiopathophysiology, and prognosis of mental health conditions in children and adolescents. This progress has been facilitated by qualitative and quantitative research studies. In the ongoing development of these topics, the current hope is that even greater advancements will be achieved by applying artificial intelligence to analyse large datasets [1].

In contrast, the evidence base for interventions, particularly those not as straightforward as medication, remains and will remain much weaker due to even much greater complexity. This translates into a significant burden for new professionals entering the field of psychotherapy, as a large proportion of families seeking help certainly want the most optimal intervention after a diagnosis has been made. Entry-level professionals in the field are confronted with a seemingly insurmountable amount of therapeutic learning material. Some of it is conceptually challenging or difficult to grasp due to its sheer volume and complexity. Moreover, some content may not be documented at all or may be presented in a poorly readable format.

Of course, in child and adolescent psychiatry, entry-level professionals can acquire their specific expertise regarding the best intervention for each individual case from various sources. In addition to reading relevant literature, attending workshops and seminars is also an excellent option. However, as in all disciplines where standardisation of intervention procedures is particularly challenging due to their high complexity and variability, practice and reflection under supervision are particularly important building blocks for education and professional development. Similar to medical disciplines that demand/emphasise manual skills, here, one can make use of the old-fashioned term of “medical art”. Unlike surgical specialties, where instructors can directly observe and guide the skills and progress of the surgeon, direct on-site supervision during psychotherapy sessions is less common. This is due to several reasons. While demonstrating techniques and providing verbal feedback is feasible in a surgical setting, it is impractical and potentially disruptive to the therapeutic relationship in psychotherapy.

Throughout their careers, both trainers and trainees across all disciplines, including child and adolescent psychiatry, turn to textbooks and scientific articles as essential resources for lifelong learning. As mentioned above, the quality and usability of this information varies depending on the topic and its grounding in scientific research. Unfortunately, the inherent complexities of the predominantly multi-modal psychiatric treatment of children, adolescents, and their families lead to the above-mentioned problems with regard to the available literature on psychotherapeutic treatment, evident even in established guidelines. These problems are exemplified by the current issue of ECAP. While presenting 30 intriguing scientific articles, only four [2–5] even mention psychotherapy in a broader sense, and just one explicitly addresses the topic of psychotherapy of depression in children and adolescents [6]. Notably, this sole article is a qualitative, not quantitative study, further highlighting the existing knowledge gap.

The four studies attempt (more or less explicitly) to demonstrate the relevance of the results of their quantitative analyses for at least partially psychotherapeutic treatments of children and adolescents. However, most such articles limit this discussion to one or two rather general sentences. Examples include: “….[to] provide new opportunities for conceptualising the integration of behavioural therapies with child mental health therapies in addressing challenging behaviour in autism…” [5] or “…This in turn impacts the therapeutic alliance, a robust mediator of treatment outcome…” [4].

In light of the situation outlined above, immense credit is due to the authors of the article recently published in ECAP “Mediators of outcome in adolescent psychotherapy and their implications for theories and mechanisms of change: a systematic review” [6]. They meticulously reviewed all randomised controlled trials investigating mechanisms of change in the psychological treatment of adolescents to identify the most promising age-, disorder- or treatment-specific mediators. Several mediators were identified to be promising for future investigations: changes in negative thoughts, dysfunctional beliefs and metacognitive skills, family functioning and parenting skills, as well as successful engagement in therapeutic activities and increased impulse control. However, naturally, this article remains on a meta-level, making it difficult for entry-level professionals to directly translate these findings into their daily practice. Furthermore, the authors themselves acknowledge that their review suggests that “…adolescent models of psychotherapy would do well to adopt a developmental perspective which takes into account the many ways in which adolescent psychotherapy differs from the psychotherapy of adults, including the factors and mechanisms that may influence therapeutic change…”. This also applies to the psychotherapeutic treatment of children. In addition, in their conclusions, they discuss the extent to which the benefits of taking a transtheoretical and transdiagnostic approach are worthwhile, as researchers can learn from different models about the variety of changes that can be observed in the process of psychotherapy. Precisely this tension between positive learning effects through transtheoretical and transdiagnostic approach, analytic approaches on the one hand and theory- and diagnosis-specific approaches on the other hand, once again demonstrates the endless number of factors and thus the complexity of successful psychotherapeutic treatment for children and adolescents.

In summary, it can be concluded that studies that investigate the classification, diagnosis, etiopathophysiology, and prognosis of child and adolescent mental health conditions typically feature larger sample sizes, greater methodological uniformity, and a more focused set of variables compared to research on psychotherapeutic interventions for this population. Consequently, the use of artificial intelligence for a comprehensive, cross-cutting analysis of studies on psychotherapeutic treatments in children and adolescents is unlikely to be successful in the foreseeable future due to the challenges in achieving sufficiently large sample sizes. Key reasons for this include the significantly higher effort required to conduct such studies, the greater difficulties in standardisation, and the more stringent selection of study participants.

In this context, it is important to mention a critical but fully under-discussed issue: unlike adults who typically seek or consent to psychotherapy, children are frequently placed in treatment at the insistence of parents or professionals, sometimes against their initial wishes or, in extreme cases, even against their will. For example, a child with anorexia nervosa may lack awareness of their illness, or a teenager with early, chronic cannabis use might resist intervention. However, especially for such scenarios, there is an urgent need for studies on the effective factors, unwanted effects, and other aspects of psychotherapeutic treatment regimens in order to support the few existing articles [7–9] with evidence from research findings, and especially to enable entry-level professionals to quickly develop effective psychotherapeutic skills and diminish their uncertainties.

In line with our recent editorial on “eXplainable Artificial Intelligence (XAI)” [1] it remains essential to combine artificial intelligence with human expertise in order to achieve interpretable results. XAI will provide us with novel and important tools and we should be open to use them as both clinicians and researchers. However, comprehending the complex realities in child and adolescent psychiatry cannot be outsourced. We excel in our work best when we combine being sophisticated and well trained clinical scientists, learning from patients, colleagues and the academic literature, while being always keen to make use of the most recent techniques.

## References

[1] Roessner V, Rothe J, Kohls G (2021). Taming the chaos?! Using eXplainable Artificial Intelligence (XAI) to tackle the complexity in mental health research. Eur Child Adolesc Psychiatry.

[2] Huang L, Cao Y, Liu Y (2023). Peer effects on rural children’s depression: do online games matter?. Eur Child Adolesc Psychiatry.

[3] Fu R, Huebner ES, Tian L (2023). Sex-specific joint trajectories of deviant peer affiliation and externalizing problems from middle childhood to early adolescence: the predictive role of family maltreatment. Eur Child Adolesc Psychiatry.

[4] van Langen MJM, van Hulst BM, Durston S (2023). Hidden in plain sight: how individual ADHD stakeholders have conflicting ideas about ADHD but do not address their own ambivalence. Eur Child Adolesc Psychiatry.

[5] Nuske HJ, Young AV, Khan FY (2024). Systematic review: emotion dysregulation and challenging behavior interventions for children and adolescents on the autism spectrum with graded key evidence–based strategy recommendations. Eur Child Adolesc Psychiatry.

[6] Rayapoullé A, de Chassey M, Benoit L (2023). Constructing childhood depression: a qualitative study with international experts in child psychiatry. Eur Child Adolesc Psychiatry.

[7] Kallas K, Toros K, Falch-Eriksen A (2021) Building a collaborative relationship with an involuntary parent in child protective services. Soc Work Werk 58. 10.15270/58-2-1040

[8] Li WHC, Xia W, Ho LLK (2021). A medical peer-delivered intervention comprising brief motivational interviewing via instant-messaging interaction to reduce drug misuse among youth in Hong Kong: a protocol for a randomised controlled trial. Addict Sci Clin Pract.

[9] Nasstasia Y, Baker AL, Lewin TJ (2019). Differential treatment effects of an integrated motivational interviewing and exercise intervention on depressive symptom profiles and associated factors: a randomised controlled cross-over trial among youth with major depression. J Affect Disord.

